# Waste Conversion into *n*-Caprylate and *n*-Caproate: Resource Recovery from Wine Lees Using Anaerobic Reactor Microbiomes and In-line Extraction

**DOI:** 10.3389/fmicb.2016.01892

**Published:** 2016-11-24

**Authors:** Leo A. Kucek, Jiajie Xu, Mytien Nguyen, Largus T. Angenent

**Affiliations:** Department of Biological and Environmental Engineering, Cornell University, IthacaNY, USA

**Keywords:** *n*-octanoic acid, *n*-hexanoic acid, *n*-caprylic acid, *n*-caproic acid, chain elongation, wine lees, carboxylate platform, reactor microbiome

## Abstract

To convert wastes into sustainable liquid fuels and chemicals, new resource recovery technologies are required. Chain elongation is a carboxylate-platform bioprocess that converts short-chain carboxylates (SCCs) (e.g., acetate [C2] and n-butyrate [C4]) into medium-chain carboxylates (MCCs) (e.g., n-caprylate [C8] and n-caproate [C6]) with hydrogen gas as a side product. Ethanol or another electron donor (e.g., lactate, carbohydrate) is required. Competitive MCC productivities, yields (product vs. substrate fed), and specificities (product vs. all products) were only achieved previously from an organic waste material when exogenous ethanol had been added. Here, we converted a real organic waste, which inherently contains ethanol, into MCCs with n-caprylate as the target product. We used wine lees, which consisted primarily of settled yeast cells and ethanol from wine fermentation, and produced MCCs with a reactor microbiome. We operated the bioreactor at a pH of 5.2 and with continuous in-line extraction and achieved a MCC productivity of 3.9 g COD/L-d at an organic loading rate of 5.8 g COD/L-d, resulting in a promising MCC yield of 67% and specificities of 36% for each n-caprylate and n-caproate (72% for both). Compared to all other studies that used complex organic substrates, we achieved the highest n-caprylate-to-ncaproate product ratio of 1.0 (COD basis), because we used increased broth-recycle rates through the forward membrane contactor, which improved in-line extraction rates. Increased recycle rates also allowed us to achieve the highest reported MCC production flux per membrane surface area thus far (20.1 g COD/m^2^-d). Through microbial community analyses, we determined that an operational taxonomic unit (OTU) for Bacteroides spp. was dominant and was positively correlated with increased MCC productivities. Our data also suggested that the microbiome may have been shaped for improved MCC production by the high broth-recycle rates. Comparable abiotic studies suggest that further increases in the broth-recycle rates could improve the overall mass transfer coefficient and its corresponding MCC production flux by almost 30 times beyond the maximum that we achieved. With improved in-line extraction, the chain-elongation biotechnology production platform offers new opportunities for resource recovery and sustainable production of liquid fuels and chemicals.

## Introduction

To provide sustainable fuels, fertilizers, and other chemicals, a paradigm shift is proposed toward resource recovery from wastes (Guest et al., 2009). Resource recovery technologies have been developed to treat many food processing and agricultural wastes (Angenent et al., 2004; Agler et al., 2011), including winery wastes (Makris et al., 2007; Mosse et al., 2011; Riaño et al., 2011). Wineries produce a variety of wastes, ranging from solids-rich wastes, such as pomace and lees (Pérez-Serradilla and de Castro, 2008), to less concentrated wastewaters (Bustamante et al., 2005). Wine lees is a common waste from wineries, which is generated during both primary and secondary decanting, and consists primarily of settled yeast cells and residual ethanol (Devesa-Rey et al., 2011). Wine lees and other winery wastes would pose ecological risks if discharged without treatment because they contain high concentrations of complex organic molecules and reduced carbon (e.g., ethanol), resulting in a considerable COD. On the other hand, these same qualities suggest that winery wastes are promising candidate substrates for the production of sustainable fuels and chemicals *via* the carboxylate platform (Agler et al., 2011; Angenent et al., 2016).

The most widely adopted carboxylate platform technology is anaerobic digestion. Anaerobic digestion is used in a variety of industries to treat diverse wastes, including winery wastes (Moletta, 2005). Anaerobic digesters employ reactor microbiomes (open cultures of microbial consortia) to convert complex organic wastes into methane-rich biogas, which is a reliable source of renewable electricity (Weiland, 2006). Reactor microbiomes can be shaped for various processes and products because they are reproducible and predictable (Werner et al., 2011, 2014; Vanwonterghem et al., 2014). In anaerobic digesters, reactor microbiomes hydrolyze complex molecules into monomers during the first step of a four-step anaerobic food web. These monomers are then converted into SCCs (which range from two to five carbons) during a second step. The carboxylate platform derives its name from SCCs because these carboxylates serve as intermediates prior to final product formation during secondary fermentation (Holtzapple and Granda, 2009; Agler et al., 2011). We use the term carboxylates here to refer to the combination of dissociated carboxylates and the corresponding undissociated carboxylic acids. During the third step of the food web, *n*-propionate, lactate, and *n*-butyrate are further broken down into acetate. The final step consists of methane production from acetate or hydrogen and carbon dioxide (Angenent et al., 2004). Without subsidization or other governmental policies, the economic viability of anaerobic digestion is often limited by the relatively low value of methane-rich biogas (Zaks et al., 2011). Therefore, to produce higher-value liquid fuels and chemicals, several researchers are expanding the carboxylate platform by developing chain elongation biotechnology processes (Angenent et al., 2016).

Chain elongation of SCCs proceeds *via* the reverse β-oxidation pathway. This pathway had been described after studying *Clostridium kluyveri*, which was recently reviewed by Spirito et al. (2014). Instead of converting SCCs into methane *via* several steps, SCCs can be chain elongated by two carbons per cycle to MCCs (which range from six to 12 carbons) (Xu et al., 2015). The yields (product vs. substrate fed) and specificities (product vs. all products) for even-chained MCC production are higher than for uneven-chained MCCs (e.g., *n*-heptanoate) (Grootscholten et al., 2013b). Therefore, we focus on *n*-caprylate and *n*-caproate production. Applications in which these MCCs can be used include: as antimicrobial agents to reduce antibiotic use in agriculture (Desbois, 2012); as fragrance and flavor intermediates (Kenealy et al., 1995); and as renewable precursors for diesel (Levy et al., 1981) and aviation fuels (Harvey and Meylemans, 2014). Longer-chain products (e.g., *n*-caprylate instead of *n*-caproate) are expected to be more valuable due to their increased hydrophobicity and energy density (Van Eerten-Jansen et al., 2013). As a result, we seek to use the reverse β-oxidation pathway to produce the longest chains possible (Angenent et al., 2016).

The reverse β-oxidation pathway requires carbon, energy, and reducing equivalents from specific electron donors (Thauer et al., 1968; Schoberth and Gottschalk, 1969). *n*-Caproate has been produced with reactor microbiomes from ethanol (Levy et al., 1981; Steinbusch et al., 2011; Agler et al., 2012), carbohydrates (Wang et al., 2007; Ding et al., 2010), and lactate (Zhu et al., 2015; Kucek et al., 2016a) as electron donors. Ethanol-fed bioreactors have demonstrated superior *n*-caproate productivities (Grootscholten et al., 2013c). In addition, *n*-caprylate has only been produced when ethanol was the electron donor (Angenent et al., 2016; Kucek et al., 2016b). Several ethanol-fed reactor microbiome experiments advanced the chain-elongation biotechnology platform, including: (1) some *n*-caprylate production during sustained *n*-caproate production (Steinbusch et al., 2011); (2) conversion of a complex organic substrate into MCCs by including an in-line extraction system (Agler et al., 2012); (3) high MCC productivities (127.5 g COD/L-d) with a short HRT (4 h) (Grootscholten et al., 2013c); (4) addition of exogenous ethanol to promote the production of MCCs from organic wastes (Grootscholten et al., 2013a); (5) and production of primarily *n*-caprylate at high productivities (19.4 g COD/L-d) by using a high ratio of ethanol to acetate (15 g COD/g COD) (Kucek et al., 2016b).

Here, our objective was to provide the first demonstration of the conversion of a real organic waste (i.e., wine lees) into *n*-caprylate and *n*-caproate without exogenous ethanol. We utilized a reactor microbiome as the inoculum for this study, which had already been shaped in a bioreactor that was fed a complex organic substrate (i.e., yeast fermentation beer from the corn-to-ethanol industry – not a waste). In addition, we tested whether increased bioreactor broth-recycle rates through the forward membrane contactor of the in-line extraction system could lead to improved undissociated MCCA extraction rates, and consequently, improved MCC production. Finally, we monitored the reactor microbiome during a phase of the operating period that included the increase in the broth-recycle rates.

## Materials and Methods

### Substrate and Inoculum

We obtained ∼75 L of wine lees during one collection from Sheldrake Point Winery (Ovid, NY, USA). The winemaker described the lees as “gross settling yeast lees from the initial fermentation,” which represents ∼4% of the volumetric production of their wine (personal communication). We measured an ethanol concentration of 180.5 g COD/L (11% v/v, 86.5 g/L, 1.88 M) (Supplementary Table S1). Wine lees consisted of 40% ethanol on a COD basis. This results in an ethanol-to-non-ethanol substrate ratio of 0.7 (COD basis). The wine lees was thoroughly mixed prior to distribution into ∼1-L containers with subsequent storage at -20°C. After thawing and before feeding to the bioreactor, we diluted the wine lees with deionized water to achieve desired substrate loading rates for each operating period (Supplementary Table S2). Finally, we adjusted the diluted substrate from pH 3.7 to 5.2 with sodium hydroxide (5 M). The inoculum was derived from a bioreactor that was fed ethanol-rich yeast fermentation beer (Agler et al., 2012). This inoculum had been fed semi-continuously (once every 2 days) throughout an operating period of ∼4 years (Ge et al., 2015).

### Experimental Periods

During three phases throughout a period of 130 days, we fed diluted wine lees to a reactor microbiome in different ways. We refer to these three phases as: batch (Phase I, Period 1, Days 0–17); semi-continuous substrate addition (Phase II, Periods 2–5, Days 17–84); and continuous substrate addition (Phase III, Periods 6–9, Days 84–130) (Supplementary Table S2). To start Phase I, we added 0.2 L of non-washed inoculum, 0.2 L of undiluted wine lees, and 4.1 L of deionized water to the bioreactor. Next, we adjusted the bioreactor broth to pH 5.2. We added fresh substrate on Days 3 and 15 in an attempt to increase the bioreactor broth concentrations of undissociated *n*-caprylic acid. Throughout Phases II and III, we continuously extracted undissociated MCCA products *via* the in-line membrane-based liquid-liquid extraction (pertraction) system. During Phase II, we fed 1 L of substrate every other day, resulting in an HRT of 9 days and a total OLR of 6 g COD/L-d. We also increased the broth-recycle rate from 9 L/d in Period 2 to 323 L/d in Period 4, and we then decreased this recycle rate to 228 L/d in Period 5 (Supplementary Table S2). During Phase III, we maintained a consistent broth-recycle rate of 228 L/d and an HRT of ∼1 day. We varied the OLR by adjusting the proportion of deionized water added to wine lees in the substrate reservoir. The OLR was increased from 8 to 52 g COD/L-d from Period 6 through Period 8, and we decreased the OLR to 39 g COD/L-d in Period 9.

### Bioreactor System

**FIGURE 1:**
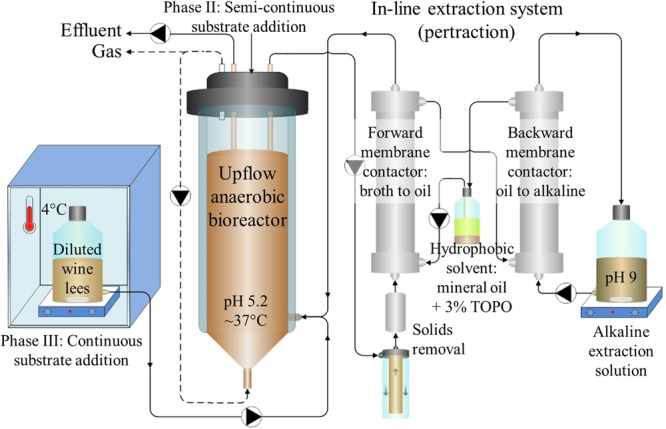
**Bioreactor system schematic.** Diluted wine lees was fed to an upflow anaerobic reactor microbiome. In-line extraction *via* a membrane-based liquid-liquid extraction system (pertraction) was used to continuously recover hydrophobic, undissociated MCCAs from a bioreactor broth recycle flow through the forward membrane contactor. After intermediary recovery in a mineral oil solvent, MCCAs were then transferred across a second, backward membrane contactor to an alkaline extraction solution. Through automatic base addition to the alkaline extraction solution, the pH gradient was maintained, and these products accumulated in the alkaline extraction solution as medium-chain carboxylates (MCCs). Adapted from Kucek et al. (2016a).

A glass bioreactor with an inner diameter of 12-cm was initially operated at a working volume of 4.5 L (Usack and Angenent, 2015) under anaerobic conditions (**Figure 1**). For temperature control, we recirculated heated water from a water bath (37 ± 1°C) (Fisher Scientific Isotemp Heated Immersion Circulator 4100C, Waltham, MA, USA) through the glass jacket surrounding the bioreactor. A pH probe (Mettler 405-DPAS SC K85, Columbus, OH, USA) was mounted through the bioreactor head plate. Automated pH control of the bioreactor broth was maintained at pH 5.2 with a controller (Eutech Instruments alpha-pH800, Vernon Hills, IL, USA) and a corresponding acid addition pump (Cole-Parmer L/S Digital Economy Drive, Vernon Hills, IL, USA). When the controller provided hydrochloric acid (0.5 M) to the bioreactor, it also initiated automated headspace gas recirculation (Mityflex 913, Bradenton, FL, USA) through the bottom of the bioreactor to enhance mixing and prevent excessive acid addition. Several glass spheres (1.8-cm diameter) were placed in the cone at the bottom of the bioreactor (del Agua et al., 2015). The biogas outlet was connected to a flow meter (Actaris Meterfabriek 1L, Delft, The Netherlands). As part of the gas collection system, we included a gas-sample septum, a bubbler, and a two-bottle water equalization system to prevent air intrusion (Ge et al., 2015).

The continuous bioreactor broth recycle (Cole-Parmer L/S Digital Economy Drive, Vernon Hills, IL, USA) proceeded first through an in-line screening and filtration system and then continued through an in-line pertraction system that is described below. During the initial 58 days only (Periods 1–2), we augmented this broth recycle with an additional flow (140 L/d) that did not pass through the in-line screen, filtration, and pertraction systems to increase mixing inside the bioreactor. The upflow hydraulic velocities in the bioreactor were similar (0.5–1.2 m/h in Supplementary Table S2) to typical values for upflow anaerobic sludge blanket (UASB) reactors (0.7–1.0 m/h) (Angenent and Sung, 2001). To maintain an HRT of 9 days, 1 L of diluted wine lees was added through a feed line in the head plate every 2 days during Phase II. When substrate was added continuously (Phase III), diluted wine lees was fed into the well-mixed recycle return near the base of the bioreactor using a peristaltic feed pump (Cole-Parmer L/S Digital Economy Drive). Diluted wine lees in the 4.2-L substrate reservoir was refrigerated (4°C) and continuously stirred, and the average rate of addition was 4.0 L/d (HRT = 1 d). Prior to collecting weekly samples for solids, COD, and microbial community analyses, we used an internal impeller (6-cm Lightnin A-310, Rochester, NY, USA) to thoroughly mix the bioreactor (200 rpm, 10 min). We also used the impeller every other day to completely mix the bioreactor contents before we removed eﬄuent and after we added substrate during Phase II (Days 17–84). During Phase III, we withdrew eﬄuent from the gas-liquid interface at the top of the bioreactor (Cole-Parmer L/S Digital Economy Drive).

### In-line Pertraction System

We used an in-line pertraction system with forward and backward membrane contactors (1.4 m^2^ each, Membrana Liqui-Cel 2.5x8, X50 membrane, Charlotte, NC, USA) (**Figure 1**), according to Kucek et al. (2016a). We recycled the bioreactor broth through the shell (exterior) side of the forward membrane contactor with a peristaltic pump (Cole-Parmer 7553-30). During Phase II, we varied the broth-recycle rate (9 to 323 L/d) to study its impact on in-line extraction; otherwise, all other pertraction system flow rates were held constant. To prevent membrane fouling, bioreactor broth was drawn from near the top of the bioreactor and was then pumped through: (1) a custom-built, 1.6-mm stainless-steel strainer (Danco 88886, Shorewood, IL, USA); (2) a large (10-in × 2-in) 30-μm sediment filter (General Electric FXWSC, Fairfield, CT, USA); (3) a 65-μm filter (McMaster-Carr 44205K21, Elmhurst, IL, USA); and (4) a 5-μm filter (Pentek GS-6 SED/5, Upper Saddle River, NJ, USA).

To extract undissociated MCCAs across the hydrophobic membrane, we used a hydrophobic mineral oil solvent. We added 30 g/L TOPO (Sigma Aldrich, St. Louis, MO, USA) to this hydrophobic solvent to encourage preferential extraction of the more hydrophobic undissociated carboxylic acids (e.g., undissociated MCCAs instead of SCCAs). The hydrophobic solvent was circulated continuously at a flow rate of 6 L/d (Cole-Parmer 7553-30) through the interior (lumen) of the membrane fibers in each membrane contactor (**Figure 1**). To prevent entrainment (i.e., leaking of hydrophobic solvent from the lumen across the hydrophobic membrane to the aqueous shell side), we provided enough backpressure on the shell side of each membrane contactor to maintain slight trans-membrane pressure gradients (∼7 kPa). Undissociated MCCAs were then transferred across the backward membrane contactor and accumulated as dissociated MCCs in the alkaline extraction solution. The alkaline extraction solution was recycled at a flow rate of 7 L/d (Cole-Parmer 7553-30) from a stirred container through the shell side of the backward membrane contactor (**Figure 1**). This alkaline extraction solution initially had a volume of 2 L and was buffered with 0.3 M sodium borate. It was maintained at pH 9 with automated addition of 5 M sodium hydroxide using a controller (Eutech Instruments alpha-pH800) and a corresponding base pump (Mityflex 913, Bradenton, FL, USA).

### Liquid and Gas Composition

Liquid samples (1.5 mL) were collected from the bioreactor broth recycle and the alkaline extraction solution daily or every other day. We collected two samples from the bioreactor broth-recycle line between the 5-μm filter and the forward membrane contactor, and one sample was collected from the well-mixed reservoir of the alkaline extraction solution. We determined the concentrations of carboxylates and ethanol in these samples with separate GC systems (Usack and Angenent, 2015). The concentrations of methane, carbon dioxide, and hydrogen gases were measured using another GC system (Usack and Angenent, 2015). For solids and COD characterization, we collected samples (100 mL) directly from the bioreactor broth on a weekly basis after thorough mixing with an internal impeller. Substrate samples were also mixed thoroughly before collection, and concentrations were determined *via* standard methods (Clesceri et al., 1998).

### Biomass Samples, DNA Extraction, PCR, Sequencing, and Microbial Community Analysis

Biomass samples were collected directly from the bioreactor broth at 10 time points during Phases I and II of the experimental period, as well as one sample from the inoculum and one sample from the wine lees substrate. Samples were collected in 2-mL Eppendorf tubes. These 2-mL samples were then centrifuged at 16,873 × *g* for 4 min, and the supernatants were discarded. The pelleted biomass samples were stored at -80°C until further processing. Genomic DNA was extracted using the PowerSoil DNA isolation kit (MO BIO Laboratories Inc., Carlsbad, CA, USA). Modifications to the protocol included utilization of custom bead tubes containing a mixture of 300 mg of 0.1-mm diameter and 100 mg of 0.5-mm diameter silica/zirconia beads (Hospodsky et al., 2010) and physical cell lysis with bead-beating at 3450 oscillations/min for 45 s. The DNA amplification protocol was performed according to Regueiro et al. (2015) with the following exceptions: (1) Mag-Bind RxnPure Plus magnetic beads solution (Omega Biotek, Norcross, GA, USA) were used instead of Mag-Bind E-Z Pure; and (2) only 20 ng DNA per sample were pooled instead of 100 ng. QIITA ^1^ was used for initial processing of the sequencing data. The sortmerna method was used to bin sequences into OTUs at 97% identity. Taxonomy was assigned for representative sequences selected for each OTU using the Greengenes v13.8 database from August 2013 (McDonald et al., 2012). The remaining analyses were performed in QIIME v1.9 (Caporaso et al., 2010). Singleton OTUs were removed from the dataset.

Community analysis, including beta diversity, was performed as described previously (Regueiro et al., 2015) with the following exceptions: (1) the alpha diversity was calculated using the Shannon diversity index (Shannon, 1948) rather than Chao1; and (2) the Pearson correlation coefficient was calculated for samples from Phase II with the functions cor and cor.test in the R stats package (R Core Team, 2013). At a significance level of *p* < 0.05 and *n* = 7, the relative abundance of an OTU would be positively correlated with the MCC productivity if the Pearson *r* was greater than 0.754. Heat maps were created to represent OTU relative abundances *via* the gplots package (Warnes et al., 2015).

### Calculations

We report concentrations and rates on a g COD basis to compare the reducing equivalents contained within substrates and products. The conversion factors (g COD/mol) are: ethanol, 96; acetate, 64; propionate, 112; *n*-butyrate, 160; *n*-valerate, 208; *n*-caproate, 256; *n*-heptanoate, 304; and *n*-caprylate, 352. Carboxylates leave the bioreactor *via* two main routes: extraction or washout in the eﬄuent. In the experimental section, we calculated the extraction rates for undissociated carboxylic acids by plotting the increasing amounts of individual carboxylates in the alkaline extraction solution against time. Least squares methods were then used to determine the slope and the sample standard deviation (LINEST function, Microsoft Excel). Next, we calculated the washout rates of carboxylates by multiplying the average bioreactor broth concentrations of carboxylates with the eﬄuent flow rates. Subsequently, we calculated the total production rates of carboxylates (g COD/d) by summing the extraction and washout rates. To calculate the volumetric production rates (productivities), we divided the production rates by the working volume of the bioreactor (g COD/L-d). To calculate the extraction and production fluxes (g COD/m^2^-d), we divided the rates (g COD/d) by the membrane surface area (*A*_transfer_, 1.4 m^2^) of the forward membrane contactor. Finally, we reported productivities as average values for each operating period. With exceptions noted, uncertainty was represented by 95% confidence intervals, as described previously (Kucek et al., 2016a).

In a separate comparative section, we used data from previous studies to predict and compare MCC production fluxes through the forward membrane contactor (g COD/m^2^-d). We predicted the extraction rates of undissociated MCCAs using a correlation, which we developed, between overall mass transfer coefficients (*k*, mm/d) and bioreactor broth-recycle superficial velocities (*u*, m/d). We also used reported data, including: forward membrane contactor surface areas (*A*_transfer_, m^2^); and concentrations of undissociated MCCAs in the bioreactor broth (*C*_MCCA_, g COD/L). Next, we calculated the washout rates for each study by: (1) multiplying the bioreactor volume (L) by the bioreactor broth concentration of MCCs (g COD/L); and then (2) dividing this multiplicative product (g COD) by the HRT (d). The total production rates, productivities, and fluxes were calculated as described above.

To calculate the recycle superficial velocity (*u*, m/d), we divided the broth-recycle rate by the cross sectional area of the forward membrane contactor shell (*A*_cross_ = 1.56 × 10^-3^ m^2^). When we varied the recycle superficial velocity (Phase II), we calculated the overall mass transfer coefficient (*k*) by dividing the extraction rate (*M*_MCCA_, g COD/d) by the multiplicative product of: (1) the membrane surface area (*A*_transfer_, m^2^); and (2) the concentration gradient of undissociated MCCAs (Δ*C*_MCCA_, g COD/L). This gradient represents the difference between the concentrations of undissociated MCCAs in the bioreactor broth and the alkaline extraction solution. Due to its high pH, the alkaline extraction solution contained negligible concentrations of undissociated MCCAs, which allowed us to estimate the concentration gradient as the bioreactor broth concentration of undissociated MCCAs.

## Results and Discussion

### A Real Organic Waste (Wine Lees) Was Converted by a Reactor Microbiome into *n*-Caprylate and *n*-Caproate at Promising Productivities and Specificities

**FIGURE 2:**
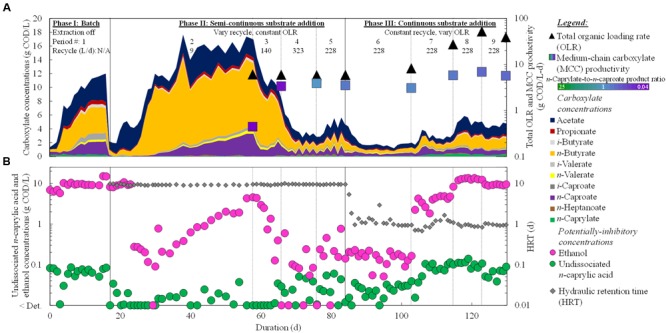
**Operating and performance parameters throughout the operating period.** Residual concentrations of carboxylates (linear scale) **(A)** and undissociated *n*-caprylic acid and ethanol (logarithmic scale) **(B)** in the bioreactor broth were plotted. Total OLRs and average MCC productivities **(A)** and HRT **(B)** were shown on logarithmic scales. The HRT was not reported for the batch phase (Phase I). A color gradient was used within the squares for the MCC productivity to show the *n*-caprylate-to-*n*-caproate product ratio (green-to-purple, respectively), with blue representing a more equal mixture of these two products. Operating phases, operating periods, and broth-recycle rates (recycle) through the forward membrane contactor of the in-line pertraction system were indicated at the top. Detection limits were 0.05 g COD/L (0.5 mM) for ethanol and ∼0.02 g COD/L (∼0.1 mM) for carboxylates.

We fed diluted wine lees to a reactor microbiome in three phases over a period of 130 days, and produced mainly *n*-caprylate and *n*-caproate (**Figure 2**). For the first time, promising MCC production was performed from a real organic waste without the addition of exogenous ethanol. During Period 8 of Phase III, we achieved a maximum MCC productivity of 6.9 g COD/L-d, but the bioreactor was overloaded at a very high OLR of 51.9 g COD/L-d (**Figure 2A**). The corresponding *n*-caprylate-to-*n*-caproate product ratio was 0.6 (COD basis) (Supplementary Table S2). Before the overloaded conditions, however, we achieved an MCC productivity of 3.9 g COD/L-d with a superior *n*-caprylate-to-*n*-caproate product ratio of 1.0 during Period 4 of Phase II (Supplementary Table S2; **Figure 2A**). Since the OLR was 5.8 g COD/L-d, we achieved an MCC yield of 67% with *n*-caprylate and *n*-caproate specificities of 36% (each) during Period 4 (Supplementary Table S2). This *n*-caprylate-to-*n*-caproate product ratio of 1.0 is the highest reported in the literature from a complex organic substrate thus far (Agler et al., 2012; Grootscholten et al., 2013a, 2014; Ge et al., 2015). Therefore, we demonstrated that equal production rates of C8 and C6 can be produced with a reactor microbiome that treats a real organic waste at an ethanol-to-non-ethanol substrate ratio of ∼0.7 (based on COD).

In a previous study, when exogenous ethanol was added to a complex organic waste (garden and food waste) in a single-stage bioprocess, the authors achieved MCC productivities up to 1.8 g COD/L-d (Grootscholten et al., 2013a). This productivity occurred after the addition of ethanol at an ethanol-to-non-ethanol substrate ratio of ∼0.1, which resulted in an *n*-caprylate-to-*n*-caproate product ratio of 0.2. Even without ethanol supplementation, some *n*-caproate was produced from innate fermentation pathways. However, *n*-caprylate production was not observed (Grootscholten et al., 2013a). Therefore, we provide the first report of *n*-caprylate production from a real organic waste without exogenous ethanol addition.

**FIGURE 3:**
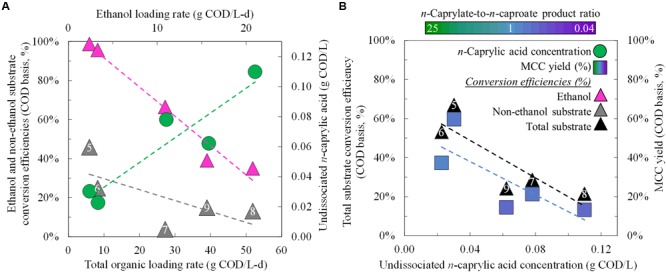
**Substrate conversion efficiencies and product yields for different operating and performance conditions during Period 5 (Phase II) and Periods 6–9 (Phase III).** Substrate conversion efficiencies and undissociated *n*-caprylic acid concentrations were plotted as a function of the total organic and ethanol loading rates **(A)**. Total substrate conversion efficiencies and MCC yields were each plotted as a function of the residual concentration of undissociated *n*-caprylic acid in the bioreactor broth **(B)**. A color gradient was used within the squares for the MCC yield to show the *n*-caprylate-to-*n*-caproate product ratio (green-to-purple, respectively), with blue representing a more equal mixture of these two products. A white font within the triangles for the non-ethanol substrate conversion efficiency and the total substrate conversion efficiency identifies the five different operating periods.

During the study by Grootscholten et al. (2013a) with garden and food waste and during Period 8 of our study with wine lees waste (Phase III), the MCC yields were low due to overloading conditions (OLRs of 72.6 and 51.8 g COD/L-d, respectively). We had increased the OLR from ∼6 to 52 g COD/L-d (Period 5 to Period 8), but this large increase of 780% only led to marginally improved MCC productivities (<70%) (**Figure 2A**; Supplementary Table S2). It also led to increased production of reduced gases in the biogas (up to 0.6 g COD/L-d), but this constituted less than 10% of the overall COD balance. The maximum hydrogen concentration in the biogas was 45% in Period 9 (Supplementary Table S2). Overloaded conditions with synthetic lactate (Kucek et al., 2016a) and ethanol (Kucek et al., 2016b) also increased the production of reduced gases. In the present study, product inhibition had restrained further increases in MCC productivities. When the OLR was increased, we observed increased concentrations of undissociated *n*-caprylic acid in the bioreactor broth (**Figure 2B**), which are known to inhibit microbial activity (Butkus et al., 2011). Importantly, average undissociated *n*-caprylic acid (C8) concentrations increased to 0.06–0.11 g COD/L (0.17–0.31 mM) during both the batch phase (Phase I) and in the phase of continuous substrate addition (Phase III) (**Figure 2B**; Supplementary Table S2). Here, the maximum undissociated *n*-caprylic acid (C8) concentration was 0.11 g COD/L (6% of its maximum solubility), which was only surpassed by our other published study (0.22 g COD/L) (Kucek et al., 2016b) wherein the undissociated *n*-caprylic acid concentration also led to product inhibition. Meanwhile, average undissociated *n*-caproic acid (C6) concentrations remained ∼10-fold lower than 2.7 g COD/L (10.5 mM) that had been observed to inhibit reactor microbiomes (Ge et al., 2015). In addition, the accumulated concentrations of ethanol during our study (∼10 g COD/L; ∼100 mM) were also lower than reported inhibitory concentrations for microbiomes and *C. kluyveri* (20–40 g COD/L; ∼200–400 mM) (Weimer and Stevenson, 2012; Angenent et al., 2016; Kucek et al., 2016b). Therefore, we postulate that these high levels of undissociated *n*-caprylic acid led to product inhibition, rather than inhibition from undissociated *n*-caproic acid or ethanol. Indeed, we found negative correlations between concentrations of undissociated *n*-caprylic acid in the broth and ethanol conversion efficiency (**Figure 3A**), overall substrate conversion (**Figure 3B**), and MCC yield (**Figure 3B**). To support chain elongation, in-line extraction can prevent undissociated MCCAs from accumulating to inhibitory levels in the bioreactor broth, which can otherwise hinder chain elongation pathways and thermodynamic feasibility.

During Period 5 and before inhibition of undissociated *n*-caprylic acids, we observed that almost all ethanol was converted (close to 100% in **Figure 3A**). This resulted in relatively low residual ethanol concentrations of 0.3 g COD/L (3.1 mM) in the broth (**Figure 2B**; Supplementary Table S2). Even without product inhibition, the conversion efficiencies for non-ethanol substrates (i.e., solids) were still low (45%) (**Figure 3A**). After, we decreased the HRT from 9.5 days (Period 5) to 1.2 days (Period 6) under similar operating conditions, the conversion efficiency of solids declined to 25% (**Figure 3A**). Clearly, the short HRTs (∼1 day) that we used during Phase III (Periods 6–9) led to insufficient residence times for hydrolysis of solids. For future work with solids-rich substrates (e.g., wine lees), we recommend a much longer retention time to promote hydrolysis. For example, for the yeast fermentation beer conversion in anaerobic fermenters, the HRT was ∼15 days (Ge et al., 2015). In our system at a low HRT of ∼1 day during Periods 6–9 (Phase III), ethanol provided the majority (>80%) of the COD that was converted into MCCs (**Figure 3A**; Supplementary Table S2). Instead of trying to convert solids at a long HRT, another opportunity would be to first remove solids and then feed a low-solids, high-ethanol substrate to the reactor microbiome at a short HRT.

### Increased Superficial Velocities within the Forward Membrane Contactor Increased Overall Mass Transfer Coefficients, Which Improved Extraction Rates, Productivities, and Product Ratios

**FIGURE 4:**
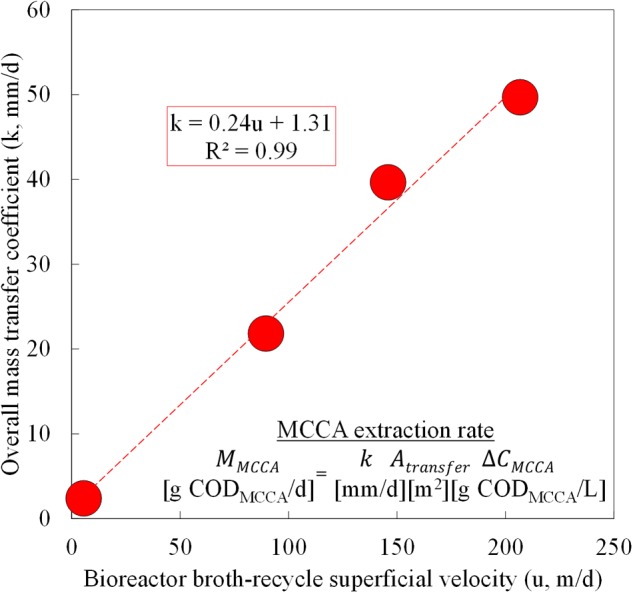
**Pertraction correlation between the overall mass transfer coefficient and broth-recycle superficial velocity through the forward membrane module during Periods 2–5.** The overall mass transfer coefficient (*k*) was plotted as function of the broth-recycle superficial velocity (*u*) for a lumped extraction of *n*-caprylic and *n*-caproic acid. These undissociated MCCAs were corrected by using g COD. The resulting k could be used in the undissociated MCCA extraction rate equation.

During Phase II (Periods 2–5; before we overloaded the reactor), we varied the broth-recycle rate through the forward membrane contactor (*A*_transfer_ = 1.4 m^2^, *A*_cross_ = 1.56 × 10^-3^ m^2^) within this biotic study to evaluate its impact on the undissociated MCCA extraction rates. Because we simultaneously extracted a mixture of undissociated MCCAs, we based the extraction rates, concentrations, and overall mass transfer coefficients on the lumped MCCA products of *n*-caprylic acid and *n*-caproic acid in g COD. We knew from previous work that increasing the recycle rates for the hydrophobic solvent or the alkaline extraction solution through the membrane contactors did not improve the undissociated MCCA extraction rates. By varying the broth-recycle rates, we confirmed a fundamental relationship that governs undissociated MCCA extraction rates: the broth-recycle superficial velocity (*u*) through the forward membrane contactor was directly proportional to the overall mass transfer coefficient (*k*) (**Figure 4**). The recycle superficial velocity (*u*) is the speed at which bioreactor broth flows past the membrane surface, and it is known that higher speeds increase turbulence and decrease resistance to MCCA transfer across the membrane (i.e., turbulence enhances extraction) (Wickramasinghe et al., 1992). Therefore, for an equivalent membrane surface area (*A*_transfer_) and undissociated MCCA concentration gradient (Δ*C*_MCCA_) (**Figure 4**), this relationship suggests that higher broth-recycle rates would improve undissociated MCCA extraction rates. Previously, an abiotic *n*-caproic acid extraction study employed a larger forward membrane contactor (*A*_transfer_ = 8.1 m^2^ and *A*_cross_ = 6.09 × 10^-3^ m^2^) and also established a linear correlation between the superficial velocity and the overall mass transfer coefficient (Kucek et al., 2016b). We observed a similar correlation for the lumped *n*-caprylic acid and *n*-caproic acid overall mass transfer coefficient under biotic conditions when compared to only *n*-caproic acid extraction under abiotic conditions (all carboxylic acids were corrected for reduced equivalents [g COD]) (Supplementary Figure S1). This indicates that the recycle superficial velocity (*u*) is directly proportional to the overall mass transfer coefficient (*k*) for Membrana Liqui-Cel X50 membrane contactors, regardless of their size.

**FIGURE 5:**
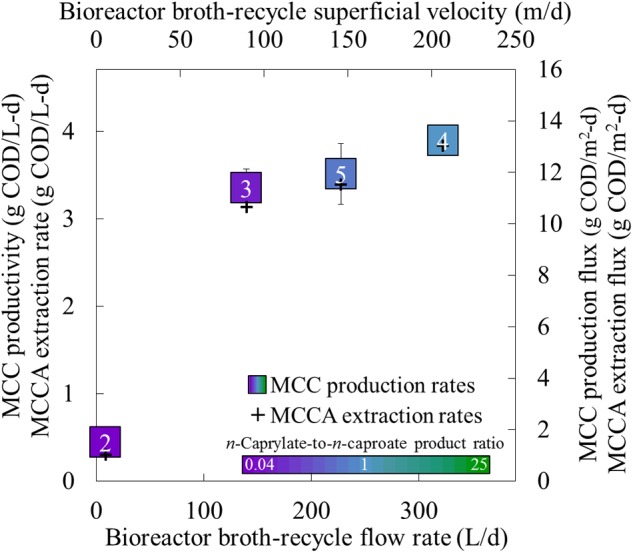
**Production performance parameters for different recycle rates in the forward membrane module during Periods 2–5.** The MCC productivity, undissociated MCCA extraction rate, MCC production flux, and undissociated MCCA extraction flux were plotted as a function of the recycle rate. The broth-recycle (flow) rate (bottom axis) and broth-recycle superficial velocity (top axis) are alternative ways to represent the same recycle rate. The square for the production rate represents the productivity (left axis) and production flux (right axis), while plus for the MCCA extraction rate represents the extraction rate (left axis) and extraction flux (right axis). A color gradient was used within the squares for the production rate to show the *n*-caprylate-to-*n*-caproate product ratio (green-to-purple, respectively), with blue representing a more equal mixture of these two products. A white font within the squares for the MCC production rate identifies the four different operating periods. Productivity uncertainty is represented by 95% confidence intervals.

By increasing the broth-recycle rate for our bioreactor, we improved the undissociated MCCA extraction rates (M_MCCA_) and fluxes by an order of magnitude (**Figure 5**). The maximum recycle superficial velocity (*u*) that we applied was 207 m/d (*k* = 50 mm/d) (**Figures 4** and **5**). From several previous abiotic membrane-based extraction experiments, much higher superficial velocities were used, which led to improved overall mass transfer coefficients (Baudot et al., 2001; Schlosser et al., 2005) (Supplementary Figure S1). A maximum superficial velocity of 6,000 m/d (6.9 × 10^-2^ m/s) was employed in a previous study, which resulted in a mass transfer coefficient of ∼4,500 mm/d (Baudot et al., 2001). Our hollow-fiber membrane contactors are designed to accommodate shell-side superficial velocities of at least 10,000 m/d. Since the present study utilized a much lower superficial velocity (below 207 m/d), new experiments should be conducted to ascertain the maximum extraction fluxes at these drastically increased superficial velocities. If the correlation between the overall mass transfer coefficient and the superficial velocity remains linear (**Figure 4**), we anticipate that an overall mass transfer coefficient of more than 1,400 mm/d could be achieved at a superficial velocity of 6,000 m/d (Supplementary Figure S1). The resulting overall mass transfer coefficient would be almost 30 times larger than what we achieved in Period 4, which should lead to proportional increases in the corresponding MCCA extraction rates.

After we increased the broth-recycle rates during Periods 2–5, we also observed increases in the MCC productivities and *n*-caprylate-to-*n*-caproate product ratios (**Figure 5**). Because of the improved MCC productivity (and improved substrate consumption), the average concentration of ethanol decreased with increased recycle rates, especially during Periods 4 and 5 when the recycle superficial velocities were above 100 m/d (Supplementary Figure S2). If we had increased the broth-recycle rate during the final phase of the experiment (Phase III), we expect that the increased extraction rates would have decreased MCCA concentrations, which would have relieved product inhibition and further improved MCC productivities. Since the operating conditions remained otherwise constant during Phase II (a fixed OLR and ethanol-to-non-ethanol substrate ratio), we postulate that improved in-line extraction rates preferentially recovered longer and more hydrophobic MCCA products from the bioreactor broth, which created a selective pressure toward continued production of longer MCC products (*n*-caprylate vs. *n*-caproate). Thus, improved in-line extraction rates would allow systems to direct substrate conversion toward increasingly hydrophobic, energy-dense, and valuable products.

**FIGURE 6:**
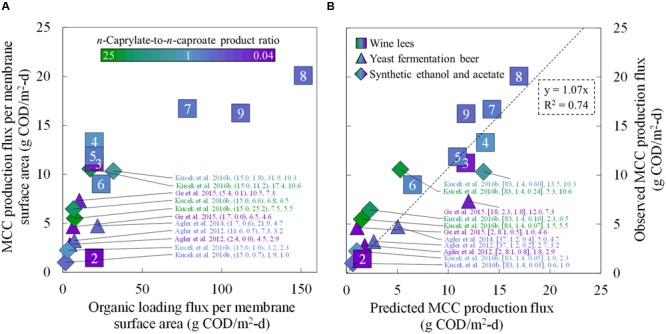
**Comparative MCC production fluxes for the present study during Periods 2–9 and for previously published studies.** Observed MCC production fluxes were plotted as a function of the organic loading flux **(A)** and as a function of the predicted MCC production fluxes **(B)**. We compared the MCC production fluxes from the present study (squares) to four reactor microbiome studies that employed in-line extraction and fed ethanol-rich substrates (triangles and diamonds) (Agler et al., 2012, 2014; Ge et al., 2015; Kucek et al., 2016b). In **(A)** the data labels include the: reference; (ethanol-to-non-ethanol substrate ratio; *n*-caprylate-to-*n*-caproate product ratio); organic loading flux; and MCC production flux. In **(B)** the data labels include the: reference; (broth-recycle superficial velocity; membrane surface area; undissociated MCCA concentration in the bioreactor broth); predicted MCC production flux, and observed MCC production flux. A color gradient was used within the squares, triangles, and diamonds for the *n*-caprylate-to-*n*-caproate product ratio (green-to-purple, respectively), with blue representing a more equal mixture of these two products. A white font within the squares for the present study identifies the eight different operating periods.

We compared the MCC production rates from several reactor microbiome studies that employed in-line extraction (**Figure 6**). To provide a common basis for comparison, we calculated the MCC production flux (g COD/m^2^-d) for each study. Here, we achieved the highest MCC flux compared to the other studies during Period 8 (up to 20.1 g COD/m^2^-d), albeit under overloaded conditions with poor MCC yields (**Figure 6A**). During an earlier period (Phase II, Period 4), we supplied a lower organic loading flux (19.9 g COD/m^2^-d), which still resulted in an MCC production flux (13.3 g COD/m^2^-d) that was higher than all previous reports (**Figure 6A**). Moreover, the *n*-caprylate-to-*n*-caproate product ratio from Period 4 (1.0 g COD/g COD) surpassed all other published studies that had used a complex organic substrate. We then proved that the correlation (*k* = 0.24*u* + 1.31 in **Figure 4**) could be used to predict the observed MCC production fluxes from several studies (**Figure 6B**). Using only reported flow rates, equipment sizes, and bioreactor broth concentrations, the predicted MCC fluxes were reasonably well correlated to the observed MCC fluxes with an *R*^2^ of 0.74 (**Figure 6B**). Higher *n*-caprylate-to-*n*-caproate product ratios (more green, up to 25 g COD/g COD) in Kucek et al. (2016b) generally led to observed MCC production fluxes that exceeded the predicted values; this is because *n*-caprylic acid is more hydrophobic and easier to extract than *n*-caproic acid. The predictive model is based on a lumped MCCA concentration parameter that was developed with comparable extraction rates of *n*-caprylic acid and *n*-caproic acid from the bioreactor broth (**Figure 4**). Therefore, this model should be further improved in future studies when *n*-caprylate comprises the majority of the MCC production.

In summary, biotic and abiotic pertraction studies with different carboxylates behaved as anticipated from previous literature studies. By increasing the broth-recycle rate through the forward membrane extraction module, we considerably improved the undissociated MCCA extraction flux (and therefore the MCC productivity) for our bioreactor setup. In addition, increased broth-recycle rates directed more substrate toward elongated carboxylate products. Future optimization studies should now be commenced to ascertain the maximum improvements that can be made with a fixed membrane surface area.

### Microbiome Analysis Revealed that Improved MCC Productivities Corresponded to a Shaped Microbial Community with Increased Abundances of *Bacteroides* spp.

**FIGURE 7:**
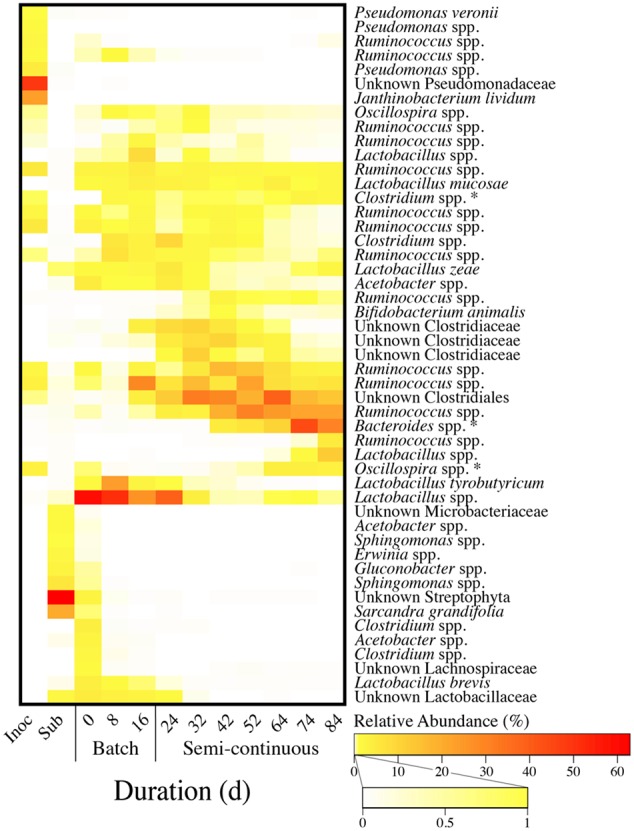
**Heatmap of relative OTU abundances for the inoculum, the substrate, and bioreactor samples from Periods 1–5.** OTUs were clustered hierarchically (average linkage) based on the Bray–Curtis dissimilarity index with sequence data for 12 microbiome samples, including: one inoculum sample; one wine lees substrate sample; three bioreactor samples from the batch phase (Period 1); and seven bioreactor samples from the phase of semi-continuous substrate addition with continuous in-line extraction (Periods 2–5). OTUs were grouped together based on both the average relative abundance and abundance profile. This resulted in grouping of OTUs with similar progressive shifts in relative abundances during the operating time. In addition, it also separated OTUs that were primarily abundant in the inoculum and the substrate. Each of the 49 OTUs listed comprised at least one percent of the relative abundance for one or more of the samples. Relative abundances of three OTUs (asterisks) were correlated (*p* < 0.05) with MCC productivities.

The microbial community was characterized based on 10 samples from the bioreactor broth during the first 84 days of this experiment (Phases I and II, Periods 1–5). In addition, we included one sample each from the inoculum and the substrate (**Figure 7**). For all 12 samples, we observed 2526 OTUs from high-quality sequence reads. For the bioreactor samples, 36 OTUs exceeded 1% relative abundance in at least one sample. For the inoculum and substrate samples, an additional six and seven unique OTUs, respectively, exceeded 1% relative abundance. The total of these 49 OTUs accounted for 86.9 to 96.6% of the total high-quality sequence reads for each sample. The number of OTUs within the community and their relative proportions (alpha diversity) did not vary considerably between microbiome samples from the operating period (Supplementary Figure S3). Moreover, the average Shannon index for bioreactor samples from the present study (3.7) was approximately equal to the average values for previous reactor microbiome studies with synthetic lactate (3.6) (Kucek et al., 2016a) or ethanol (3.4) (Kucek et al., 2016b) as electron donors.

The 49 OTUs were hierarchically ranked based on both the average relative abundance and the abundance profile throughout Phases I and II. Consequently, grouped OTUs in the heat map had similar shifts in abundances for the inoculum, the substrate, and throughout the operating time of the bioreactor (**Figure 7**). Even though the inoculum was derived from a similarly operated bioreactor that was fed ethanol-rich yeast fermentation beer, we observed that the most dominant OTUs in the wine lees-converting bioreactor were relatively scarce in the inoculum (**Figure 7**). In addition, our substrate (wine lees) contained a unique microbial community compared to the bioreactor broth samples (**Figure 7**). As an open-culture biotechnology platform, we had not attempted to modify or suppress the microbiome in this substrate. Rather, ecological selection for MCC production was achieved by operating the bioreactor under controlled environmental parameters (e.g., pH, temperature, residual bioreactor broth concentrations).

During the batch phase (Phase I), OTUs for *Lactobacillus* spp. were predominant (21.9 to 50.3%) (**Figure 7**). Next, the relative abundances of these OTUs declined after in-line extraction was initiated at the beginning of Phase II. Concurrently, we observed increases in relative abundances of the OTUs for *Ruminococcus* spp. and other unknown species of the order Clostridiales and family Clostridiaceae (**Figure 7**). The broth-recycle rate through the forward membrane contactor was increased from a very low level in Period 2 to increasingly higher levels during Periods 3 and 4, and then back down to an intermediate level in Period 5 (**Figure 5**). When recycle rates had been increased to their maximum value during Period 4, an OTU for *Bacteroides* spp. surged in relative abundance on Day 74 (**Figure 7**). Period 4 also corresponded with the highest MCC productivity and maximum *n*-caprylate-to-*n*-caproate product ratio observed in this study. Accordingly, we determined that the MCC productivity was positively correlated with the relative abundance of *Bacteroides* spp. (*p* = 0.01), as well as with two less abundant OTUs for *Oscillospira* spp. (*p* = 2.5 × 10^-5^) and *Clostridium* spp. (*p* = 0.02) (Supplementary Figure S4; **Figure 7**). In previous studies with similar inocula, we had also observed positive correlations between the MCC productivity and the relative abundance of the OTUs for *Bacteroides* spp. and *Oscillospira* spp. when the electron donors were lactate and ethanol, respectively (Kucek et al., 2016a,b).

**FIGURE 8:**
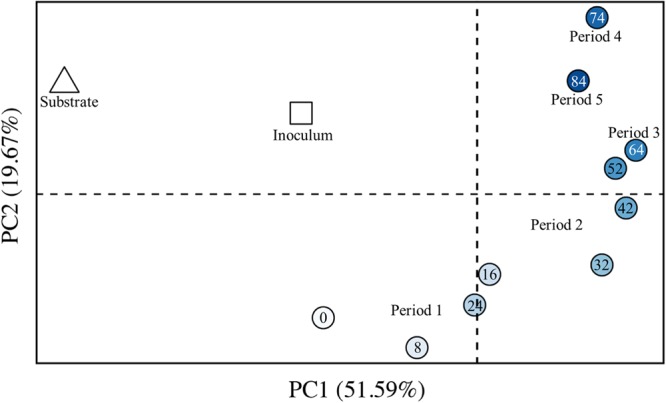
**Beta diversity for the inoculum, the substrate, and bioreactor samples from Periods 1–5.** PCoA of weighted UniFrac distances was performed with sequence data for 12 microbiome samples, including: one inoculum sample (square); one wine lees substrate sample (triangle); three bioreactor samples from the batch phase (Period 1); and seven bioreactor samples from the phase of semi-continuous substrate addition with continuous in-line extraction (Periods 2–5) – bioreactor samples are shown by circles. The increasingly darker blue circles were collected at later days. The numbers within the circles indicates the collection day number. The increased distance between samples represents increased dissimilarity. The first two PCoA axes are shown, which together explain 72% of the overall phylogenetic variation between samples; PC1 explains 52%, while PC2 explains 20% of the overall phylogenetic variation.

We also analyzed the dissimilarity of the OTU composition between pairs of samples by using the weighted UniFrac algorithm (beta diversity) (Lozupone and Knight, 2005), resulting in greater distances between more dissimilar samples. Principal coordinates analysis (PCoA) allows us to visualize these distances on a 2-dimensional plot (**Figure 8**). We observed a chronological order from early samples (lighter circles) to late samples (darker circles). One discrepancy in this chronological order was observed for Periods 4 and 5 (Days 74 and 84, respectively) (**Figure 8**). The sample from Day 84 was actually more similar to the sample from Day 64 than for the sample from Day 74. We had increased the broth-recycle rate from Period 2 through Period 4 (Day 74), and then we decreased the recycle rate during Period 5 again (Day 84) (**Figure 5**). Therefore, we suggest that the larger distance between the sample from Period 4 (Day 74) and earlier samples was due to the increased broth-recycle rate. The most dissimilar sample from our study (Day 74) represented the sample from the bioreactor with the highest broth-recycle rate, MCC productivity, and *n*-caprylate-to-*n*-caproate ratio (**Figure 5**), identifying that the microbiome had likely been shaped for its function. However, a future study would be needed to substantiate this finding.

## Conclusion

In a reactor microbiome with continuous in-line extraction, we provide the first demonstration of MCC production from a real organic waste (wine lees) without exogenous ethanol addition at a promising productivity of 7 g COD/L-d and a specificity of 36% for each *n*-caprylate and *n*-caproate (72% for both). Compared to other studies that used complex organic substrates, we also achieved the highest *n*-caprylate-to-*n*-caproate product ratio of 1.0 (based on COD). Microbial community analyses indicated that OTUs for *Bacteroides* spp., *Oscillospira* spp., and *Clostridium* spp. were positively correlated with increased MCC productivities. At the end of the study, when we increased the substrate loading, undissociated *n*-caprylic acid concentrations prevented further increases in the MCC productivity due to product inhibition. We used data from biotic and abiotic studies to suggest that increased broth-recycle superficial velocities could have further increased the MCC productivities to almost 30 times the current maximum level with a fixed membrane surface area. Our data also shows that increasing the superficial velocities will change the microbiome.

To advance the chain-elongation biotechnology platform, we encourage new in-line pertraction experiments at high bioreactor broth-recycle rates (>1,000 m/d; >1.2 × 10^-2^ m/s). These new experiments will require careful analysis (e.g., loading rates and inhibitory concentrations) and system optimization (e.g., solids removal and pressurized systems). We also encourage the development of a detailed TEA to guide technology deployment. Specifically, economic guidance is required to: (1) optimize between increased capital and operating costs; (2) select appropriate (waste) substrates; and (3) develop marketable MCC products. With continued development, we believe that chain elongation with in-line extraction can provide a new route for resource recovery and the sustainable production of liquid fuels and chemicals from wastes.

## Sequence Data

16S rRNA sequence data and metadata are available on QIITA (http://qiita.microbio.me; study ID 10517) and the EBI database (www.ebi.ack.uk, accession number: ERP016172).

## Author Contributions

LA proposed the study, and provided guidance to all co-authors. LK and JX: constructed the bioreactor system; designed and conducted the experiment; collected samples; and determined analyte concentrations. LK analyzed bioreactor system data and conducted calculations. MN performed all methods to characterize the microbial community, including corresponding data analysis and figure preparation. LK and JX prepared other figures. LA and LK wrote the manuscript, with revisions from JX and MN.

## Conflict of Interest Statement

The authors declare that the research was conducted in the absence of any commercial or financial relationships that could be construed as a potential conflict of interest.

## Footnotes

1 qiita.microbio.me

## Abbreviations

COD: chemical oxygen demand
GC: gas chromatography
HRT: hydraulic retention time
MCC: medium-chain carboxylate
MCCA: medium-chain carboxylic acid
OLR: organic loading rate
OTU: operational taxonomic unit
PC: principal coordinate
PCoA: principal coordinates analysis
SCC: short-chain carboxylate
SCCA: short-chain carboxylic acid
TEA: techno-economic analysis
TOPO: tri-*n*-octylphosphine oxide
