# Supplementary material for: Waste Conversion into n-Caprylate and n-Caproate: Resource Recovery from Wine Lees Using Anaerobic Reactor Microbiomes and In-line Extraction
Source: Front Microbiol. 2016 Nov 24;7:1892. doi: 10.3389/fmicb.2016.01892 (PMC5121217; doi:10.3389/fmicb.2016.01892)
Supplement: Supplementary file 1 [file Data_Sheet_1.DOCX]

SUPPLEMENTAL INFORMATION

for

**Waste conversion into *n*-caprylate and *n*-caproate: resource recovery from wine lees using anaerobic reactor microbiomes and in-line extraction**

**Leo A. Kucek, Jiajie Xu, Mytien Nguyen and Largus T. Angenent**

Biological and Environmental Engineering, Cornell University, Ithaca, NY 14853, USA

**Table S1. Wine lees composition.** Wine lees is a winery waste that contains ethanol (approximately 11% v/v) and other complex organic substrates. The ethanol-to-non-ethanol substrate ratio was 0.7 (COD basis). Average concentrations and ratios are presented and uncertainty is represented by 95% confidence intervals.

**
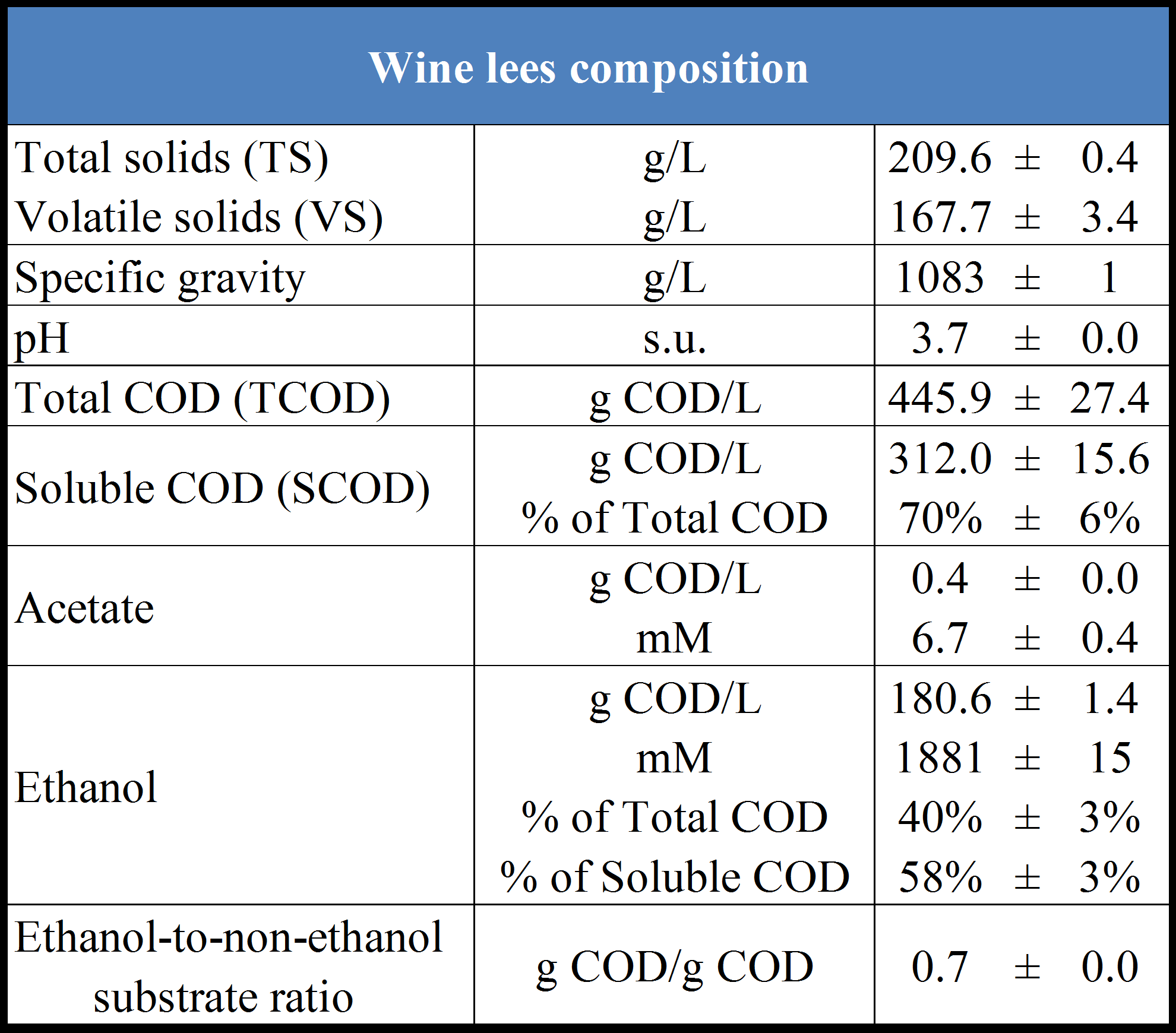
**

**Table S2. Comprehensive data table: operating conditions, bioreactor concentrations, and substrate and product rates.** We fed diluted wine lees to a reactor microbiome in three phases over a period of 130 days: in a batch phase (Phase I, Period 1, Days 0-17), semi-continuously (Phase II, Periods 2-5, Days 17-84), and continuously (Phase III, Periods 6-9, Days 84-130). Phase I was in batch operation; thus, substrate loading and production rates were not reported. Otherwise, average values are reported for each operating period. **(A)** Operating conditions included the bioreactor broth-recycle (flow) rate (r, L/d) or superficial velocity (u, m/d) through the forward membrane contactor of the in-line extraction system. The bioreactor upflow velocity included flow from this recycle rate, plus additional flow from a dedicated upflow recirculation pump during Periods 1 and 2 only (Days 0-58). **(B)** Substrate ratios were fixed at 0.7 (COD basis), but organic loading rates were varied in Phase III (Periods 6-9, Days 84-130). The concentrations of ethanol and total organics (g COD/L) in the diluted wine lees substrate can be calculated by multiplying the reported average ethanol and total organic loading rates (g COD/L-d) by the corresponding average HRT (d). Converted ethanol rates were calculated as the difference between the ethanol loading rate and the quotient of the residual ethanol concentration divided by the HRT. The total substrate conversion was calculated as the total carboxylate production rate. The non-ethanol substrate conversion was calculated as the difference between the total substrate conversion and the ethanol conversion rate. **(C)** The carboxylate productivities and *n*-caprylate-to-*n*-caproate product ratios are reported for each operating period. The total carboxylate production rates (g COD/d) were the sum of average effluent rates plus average extraction rates. Carboxylate productivities were calculated by dividing the production rates by the bioreactor working volume. **(D)** Gas production was minimal, although maximum overloaded conditions in Period 8 (Phase III, Days 115-123) led to the highest gas production in the present study. **(E)** Bioreactor broth concentrations are presented, including solids, COD, ethanol, (dissociated *plus* undissociated) carboxylates, and undissociated MCCAs. Detection limits were approximately 0.05 g COD/L (0.5 mM) for ethanol and approximately 0.02 g COD/L (~0.1 mM) for other carboxylates. **(F)** By dividing the MCC production rate (and MCCA extraction rate) (g COD/d) by the membrane surface area (A_transfer_, 1.4 m^2^), we calculated the MCC production flux and (MCCA extraction flux) (g COD/m^2^-d) for each period. Finally, the extraction efficiency was determined by dividing the MCCA extraction flux by the MCC production flux. **B.D.**: below detection; **N/A**: not available (*i.e.*, no replicates). Uncertainty is represented by 95% confidence intervals, except for gas composition, solids, and COD data, wherein the uncertainty is represented by the standard error (n = 3).


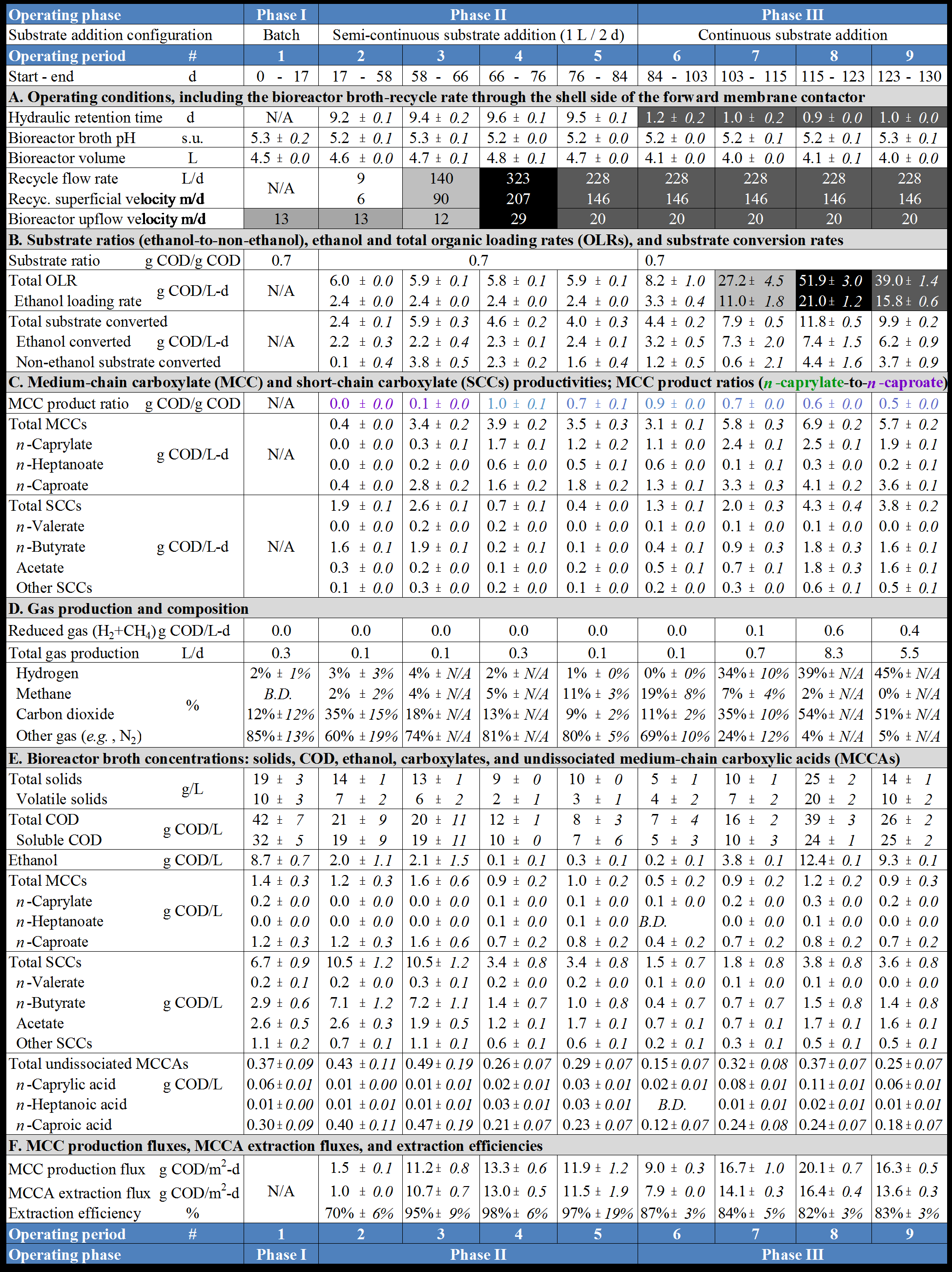


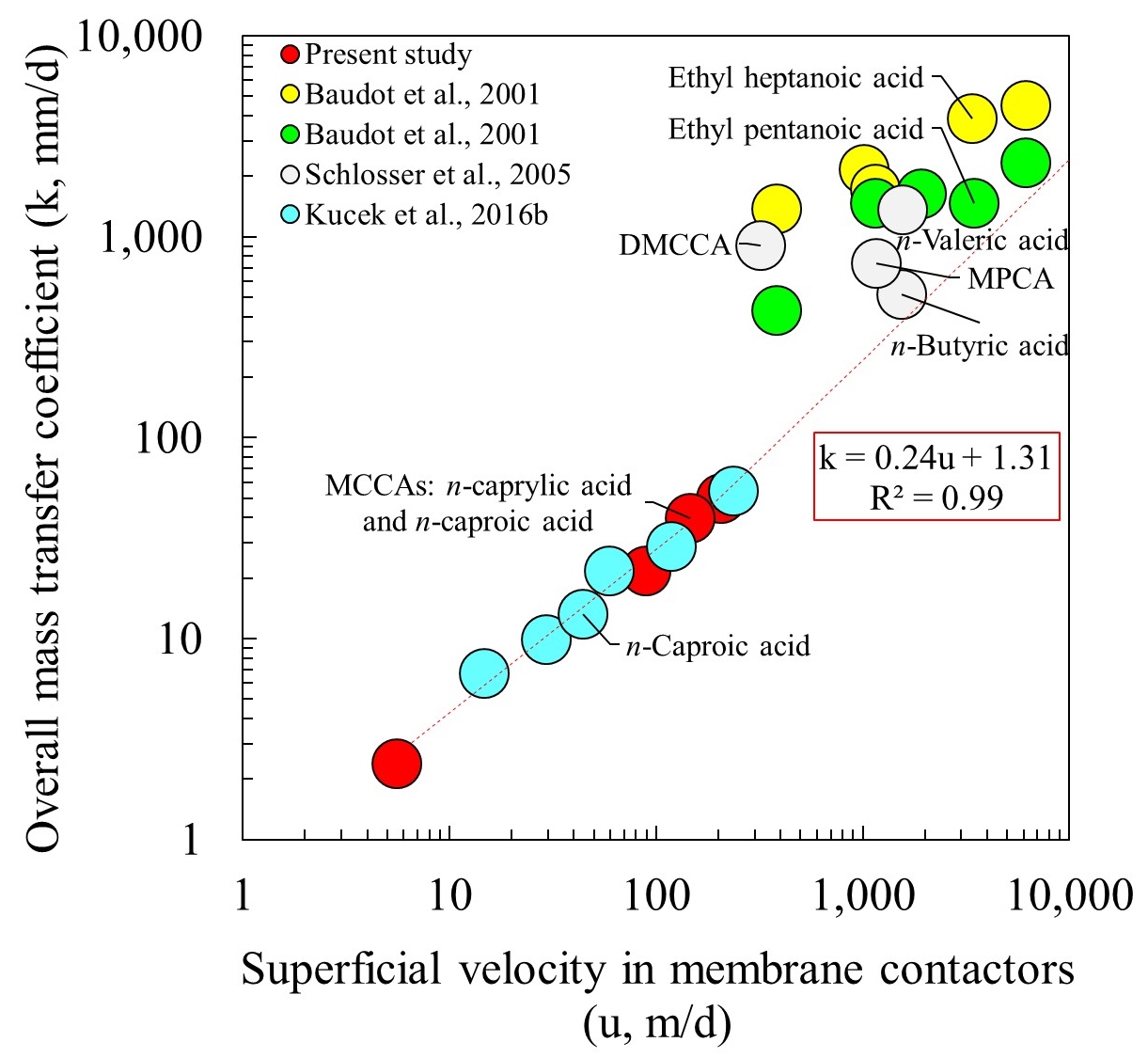


**Figure S1. Correlations between overall mass transfer coefficients and superficial velocities for varied undissociated carboxylic acids in biotic and abiotic studies.** Broth-recycle superficial velocities (u) through the forward membrane contactor and the corresponding overall mass transfer coefficients (k) were plotted on a logarithmic scale. Both the biotic data from our present study (lumped *n*-caprylic and *n*-caproic acid) and the abiotic data from a previous study (*n*-caproic acid only) (Kucek et al., 2016b) are shown, and the correlation from the present biotic data is used for the linear correlation. Literature values from two other abiotic studies that used Membrana Liqui-Cel X50 membrane contactors were also shown for different carboxylic acids (indicated) (Baudot et al., 2001;Schlosser et al., 2005). Because these studies used similar membrane contactors of varied sizes to extract comparable carboxylic acids, we anticipate that researchers could improve the overall mass transfer coefficients by one to two orders of magnitude by increasing recycle superficial velocities. If the MCCA concentration gradient (∆C_MCCA_) remained equivalent at these higher overall mass transfer coefficients, then the MCCA extraction flux would increase proportionally.


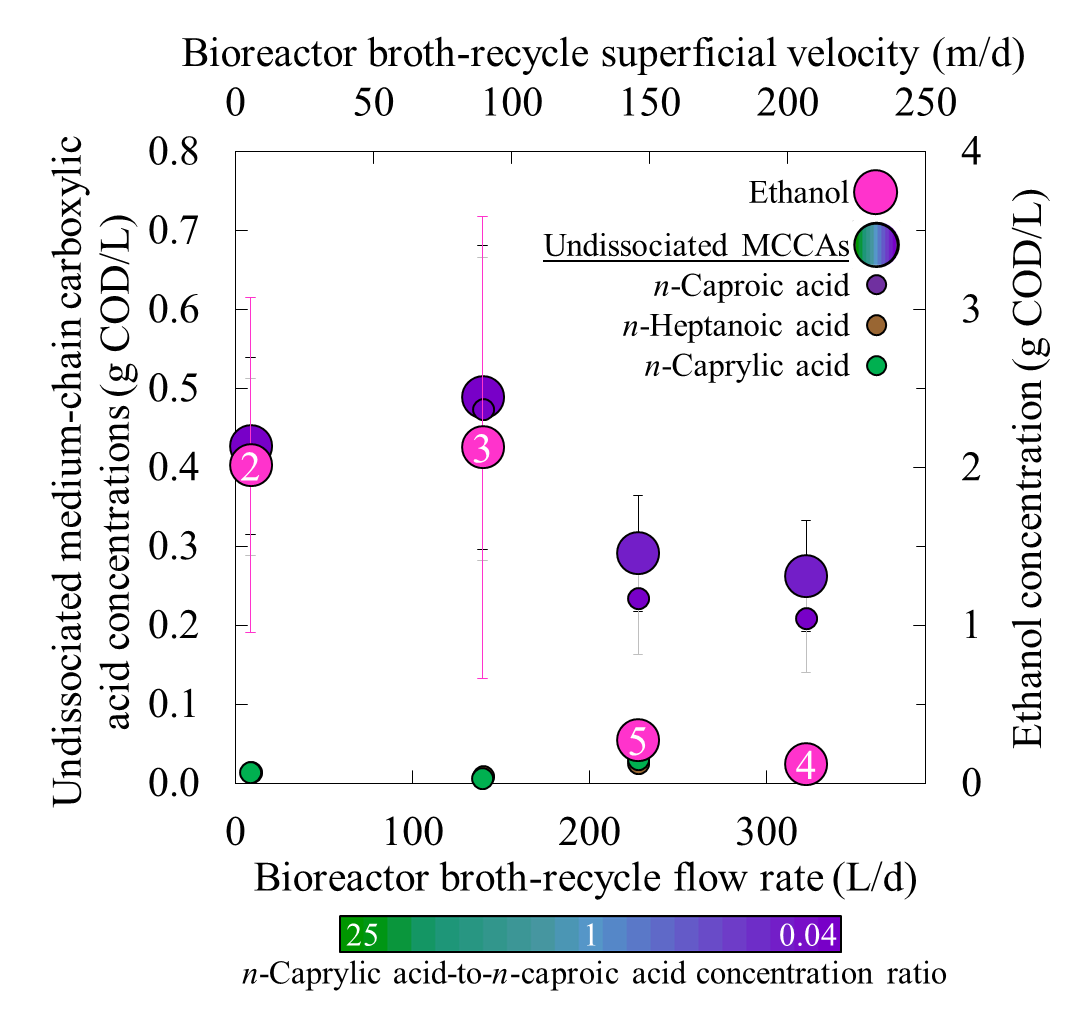


**Figure S2. Residual concentrations of undissociated MCCAs and ethanol as a function of recycle rates through the forward membrane contactor during Periods 2-5.** We varied the broth-recycle rate through the forward membrane contactor. The total undissociated MCCA concentrations (large purple circles) and individual MCCAs (small circles) were generally diminished with increased recycle rates. Average concentrations from each period are presented, and uncertainty is represented by 95% confidence intervals. A color gradient was used within the large non-pink circles for the undissociated MCCAs to show the *n*-caprylic acid-to-*n*-caproic acid concentration ratio (green-to-purple, respectively), with blue representing a more equal mixture of these two products. A white font within the pink circles for ethanol concentration identifies the four different operating periods.


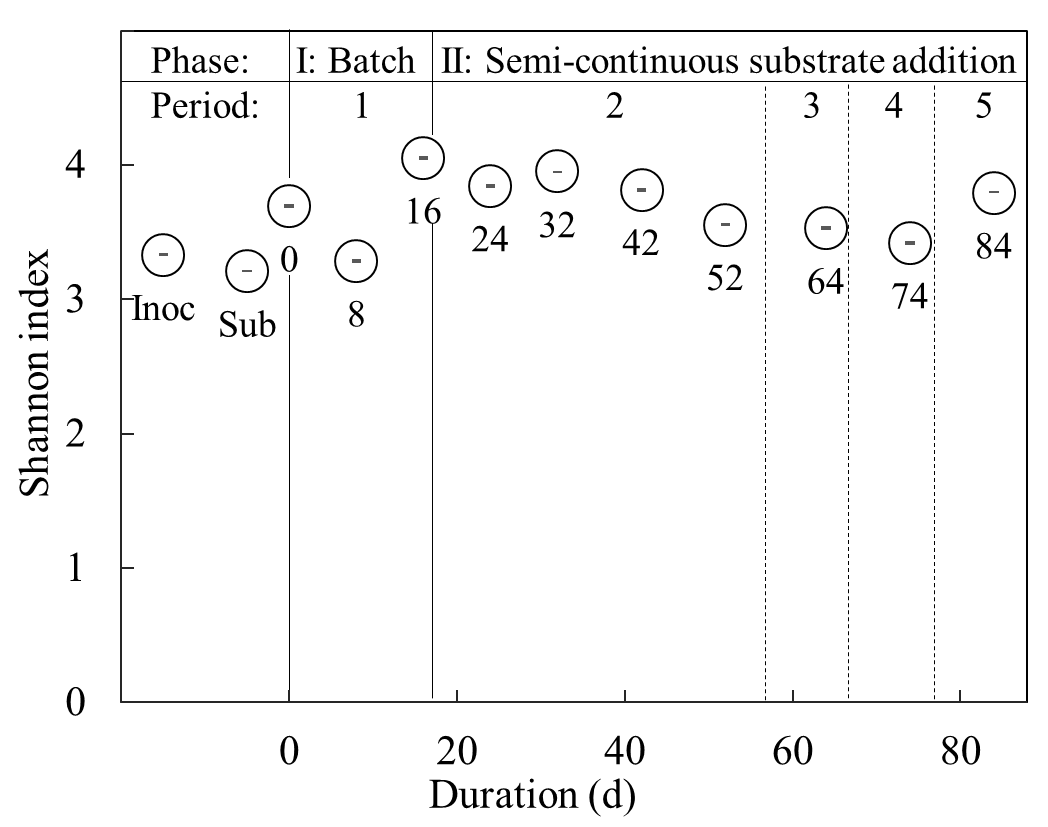


**Figure S3. Alpha diversity during Phases I and II.** Microbiome samples were collected during the batch phase (Phase I) and during the phase of semi-continuous substrate addition (Phase II) in addition to the inoculum and substrate. The Shannon index was used to determine the evenness and richness for these 12 microbiome samples. The average value of the Shannon index for all bioreactor samples was 3.7, and uncertainty is represented by 95% confidence intervals based on ten independent rarefactions.


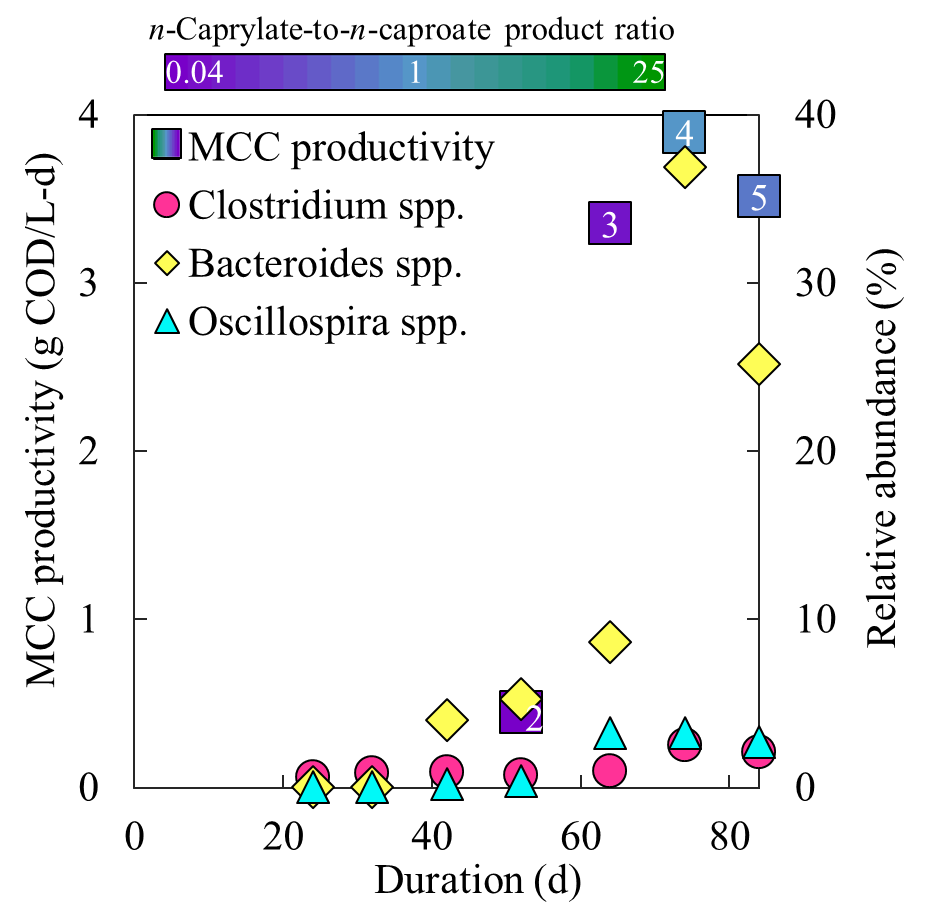


**Figure S4. Correlation between OTUs and MCC productivities during Phases I and II.** Microbiome samples were collected during the batch phase (Phase I) and during the phase of semi-continuous substrate addition (Phase II). Three OTUs were shown to correlate significantly (p<0.05) with average MCC productivities from each operating period in Phase II. A color gradient was used within the squares for the MCC productivity to show the *n*-caprylate-to-*n*-caproate product ratio (green-to-purple, respectively), with blue representing a more equal mixture of these two products. A white font within these squares identifies the four different periods during Phase II.

**REFERENCES**

Baudot, A., Floury, J., and Smorenburg, H.E. (2001). Liquid-liquid extraction of aroma compounds with hollow fiber contactor. *AIChE Journal* 47**,** 1780-1793. doi: 10.1002/aic.690470810.

Kucek, L.A., Nguyen, M., and Angenent, L.T. (2016a). Conversion of L-lactate into *n*-caproate by a continuously fed reactor microbiome. *Water Research* 93**,** 163-171. doi: 10.1016/j.watres.2016.02.018.

Kucek, L.A., Spirito, C.M., and Angenent, L.T. (2016b). High n-caprylate productivities and specificities from dilute ethanol and acetate: chain elongation with microbiomes to upgrade syngas fermentation products. *Energy and Environmental Science*, doi: 10.1039/c6ee01487a.

Schlosser, Š., Kertész, R., and Marták, J. (2005). Recovery and separation of organic acids by membrane-based solvent extraction and pertraction: An overview with a case study on recovery of MPCA. *Separation and Purification Technology* 41**,** 237-266. doi: 10.1016/j.seppur.2004.07.019.
